# Vascular Anchoring Bias in a Type A Aortic Dissection Survivor: Diagnostic Delays in Acute Care Evaluation of Hepatic and Urologic Pathology

**DOI:** 10.7759/cureus.107523

**Published:** 2026-04-22

**Authors:** Jason Park, Michail Koutentakis, Issac Park, Emmanuel Faluade, Rodolfo J Oviedo

**Affiliations:** 1 Department of Biomedical Sciences, Texas A&M University, College Station, USA; 2 College of Medicine, Texila American University, Georgetown, GUY; 3 Department of Experimental and Clinical Pharmacology, Medical University of Warsaw, Center for Preclinical Research and Technology (CEPT), Warsaw, POL; 4 Department of Intensive Care Medicine, National Medical Center, Seoul, KOR; 5 Department of Anesthesiology and Pain Medicine, Independent Scholar of Anesthesiology and Pain Medicine, Houston, USA; 6 Department of Surgery, Nacogdoches Medical Center, Nacogdoches, USA; 7 Department of Surgery, University of Houston Tilman J. Fertitta Family College of Medicine, Houston, USA; 8 Department of Surgery, Sam Houston State University College of Osteopathic Medicine, Conroe, USA

**Keywords:** anchoring bias, aortic dissection, clinical decision-making, cognitive bias, diagnostic error, focal nodular hyperplasia, healthcare systems, nephrolithiasis

## Abstract

Survivors of type A aortic dissection require lifelong surveillance and present unique diagnostic challenges when new symptoms arise. Cognitive biases, including anchoring bias, may cause medical practitioners to overprioritize vascular etiologies, thereby delaying recognition of more common non-vascular conditions.

We report the case of a 59-year-old male with a history of type A aortic dissection diagnosed in 2016 and managed conservatively with medical therapy and long-term imaging surveillance, who later presented with both a hepatic mass lesion and recurrent ureteral stones. The patient’s history of catastrophic vascular disease created a persistent anchoring bias that influenced subsequent diagnostic pathways. A 4.4 cm liver mass raised suspicion for malignancy due to its arterial enhancement pattern. This initial concern for malignancy resulted in prolonged diagnostic uncertainty. Ultimately, the mass lesion proved to be a benign lesion of focal nodular hyperplasia (FNH), diagnosed following biopsy. The acute presentation of right flank pain initially prompted evaluation for aortic complications before the diagnosis of an obstructing ureteral stone, ultimately requiring four extracorporeal shock wave lithotripsy (ESWL) procedures. Diagnostic delays were further compounded by concurrent healthcare system disruptions during a period of workforce instability in South Korea.

This case illustrates how prior catastrophic vascular disease can create durable diagnostic anchoring, delaying recognition of common non-vascular conditions. System-level disruptions further amplified this delay. Implementation of structured diagnostic approaches and cognitive forcing strategies may mitigate anchoring bias in high-risk vascular populations.

## Introduction

Type A aortic dissection, a life-threatening condition involving a tear in the ascending aorta, is one of the most lethal vascular emergencies, with an in-hospital mortality rate of 5-29% despite advances in modern management strategies [1]. It results from an intimal tear in the ascending aorta, allowing blood to enter the medial layer and create a false lumen, which may propagate and compromise branch vessel perfusion. Survivors often undergo long-term imaging follow-up due to the risk of late aortic complications [2]. The enduring clinical vigilance associated with this diagnosis may lead clinicians to reasonably prioritize exclusion of vascular complications, though this vigilance may also influence diagnostic framing when more common conditions are present.

Anchoring bias, defined as the tendency to rely heavily on initial impressions during clinical reasoning, has been described in emergency medicine settings [3,4]. In a recently performed multi-country scoping review for cognitive biases in pre- and in-hospital critical care medicine, anchoring bias was among the most frequently identified cognitive biases in pre-hospital and critical care settings [5]. When evaluating patients with a history of life-threatening vascular events, clinicians may be more likely to prioritize exclusion of vascular complications when new abdominal or chest pain arises, potentially delaying consideration of more common non-vascular conditions such as hepatic masses or urolithiasis [3-5].

We present a case of a type A aortic dissection survivor in whom diagnostic anchoring bias and system-level healthcare disruptions led to significant delays in identifying benign hepatic and urologic conditions. This case highlights the importance of structured cognitive debiasing strategies and systematic diagnostic approaches in caring for high-risk vascular populations, and it illustrates how survivorship from a life-threatening vascular event can generate a durable cognitive anchor influencing subsequent diagnostic pathways. Despite increasing awareness of diagnostic error in medicine, the impact of survivorship from catastrophic cardiovascular disease on subsequent diagnostic reasoning remains underexplored.

## Case presentation

**Figure 1:**
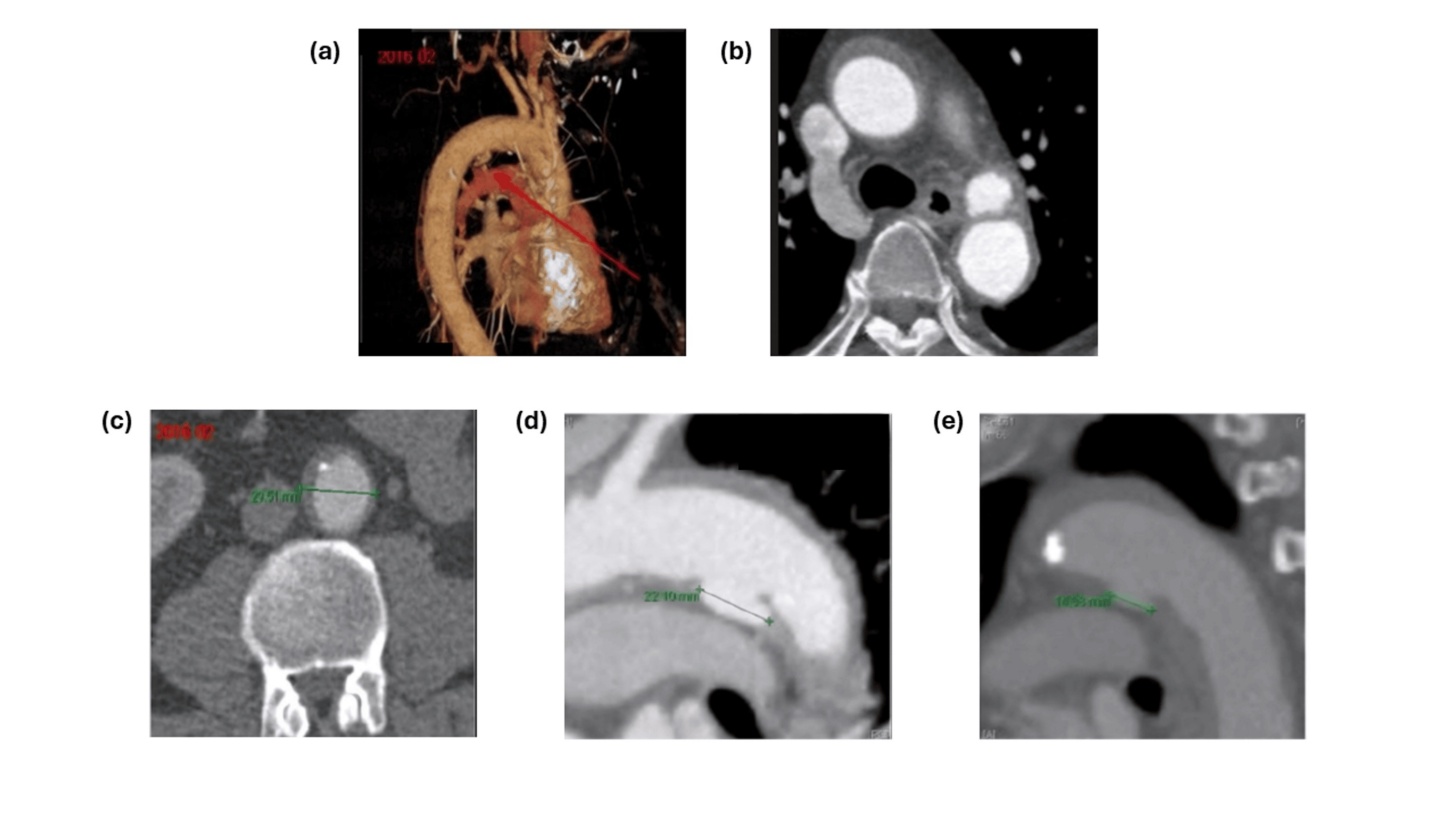
Computed tomography (CT) imaging demonstrating vascular abnormality (a) Three-dimensional reconstructed CT angiography showing the thoracic aorta. The red arrow indicates the region of type A aortic dissection-related focal dilation and structural abnormality of the aortic wall. (b) Axial contrast-enhanced CT image of the thoracic aorta demonstrating abnormal dilation with surrounding structural changes (arrow), consistent with focal vascular pathology. (c) Axial CT image showing measurement of the affected vascular segment (approximately 23.5 mm), indicating enlargement compared to the expected normal diameter. (d) Sagittal CT reconstruction demonstrating longitudinal extent of the lesion (approximately 22.1 mm), highlighting involvement of the aortic wall. (e) Additional sagittal/oblique CT view showing variation in measured diameter (approximately 14.9 mm), suggesting irregular morphology of the lesion. All images were obtained from the patient’s original medical records. Arrows indicate regions of interest corresponding to structural vascular abnormalities.

A 59-year-old male with a prior history of type A aortic dissection diagnosed in 2016 and well-controlled hypertension underwent routine surveillance imaging in 2025 at Samsung Medical Center in Korea. At the time of the initial diagnosis, the patient was managed conservatively with medical therapy and close imaging surveillance rather than surgical intervention. The patient remained asymptomatic from a cardiovascular standpoint and continued to adhere to the recommended follow-up guidelines for aortic dissection survivors (Figure 1).

**Figure 2:**
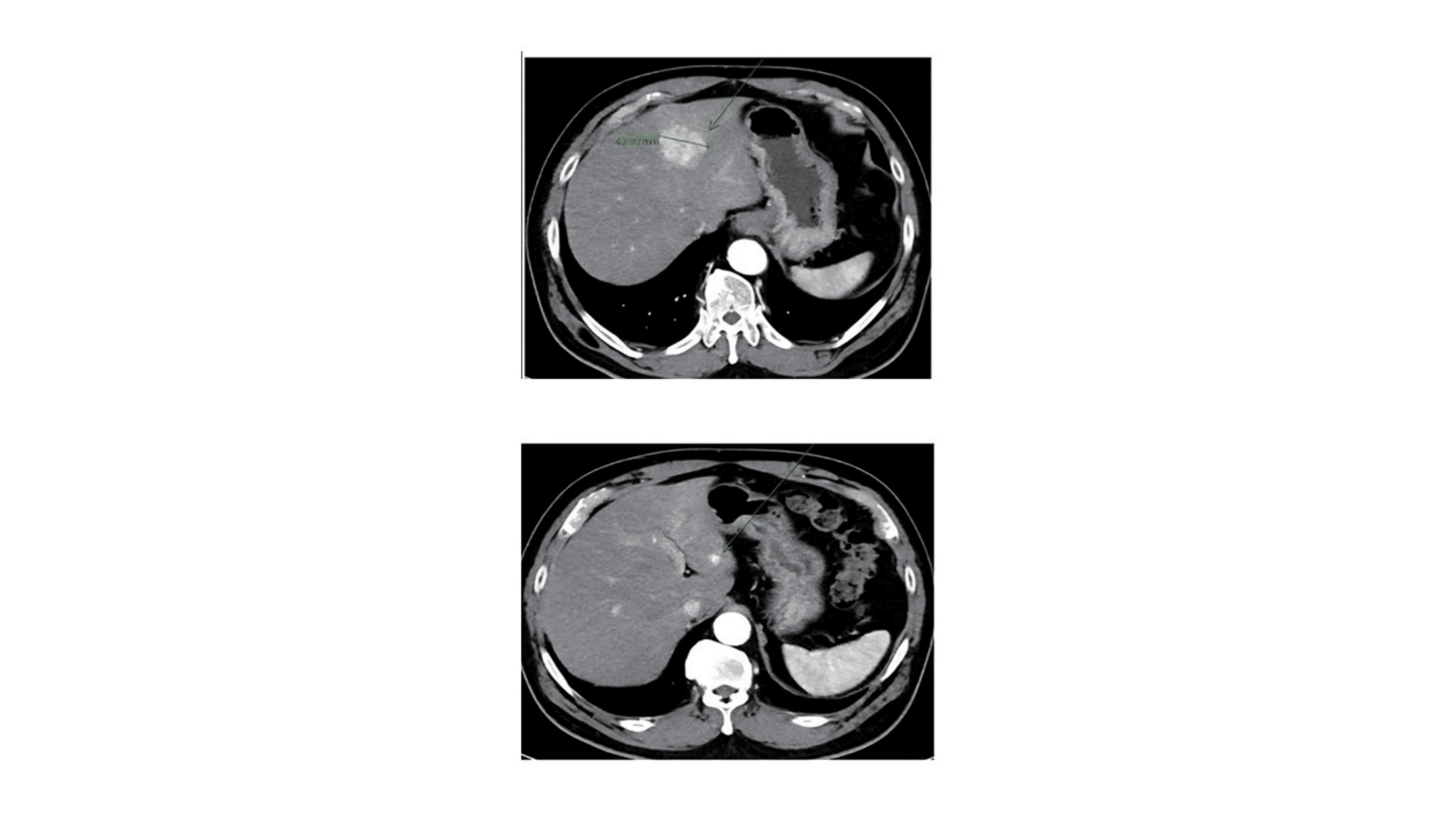
Contrast-enhanced CT demonstrating hepatic lesion Contrast-enhanced axial CT images demonstrate a 4.3-4.4 cm hyperenhancing lesion in liver segments III/IV (arrows), consistent with FNH, later confirmed by biopsy. The images were obtained directly from the patient’s diagnostic CT records and are presented in their original format. Additional imaging, including MRI and ultrasound, was not available, as MRI images are not readily accessible in the electronic medical record, and ultrasound images were not stored in the accessible system. All images have been de-identified and standardized for presentation.

Surveillance contrast-enhanced CT revealed an incidental 4.3-4.4 cm enhancing mass in segments III/IV of the liver (Figure 2). Subsequent MRI (report not shown) demonstrated arterial enhancement characteristics concerning a hepatocellular nodule. Given his vascular history, there was initial concern for possible vascular abnormalities or complications related to his prior dissection. The differential diagnoses included focal nodular hyperplasia (FNH), hepatocellular adenoma, and hepatocellular carcinoma.

Given the patient’s prior vascular history, diagnostic attention remained disproportionately focused on excluding vascular complications, which contributed to delayed prioritization of tissue diagnosis despite imaging features suggestive of a benign hepatic process.

The tissue diagnosis was significantly delayed due to healthcare system disruptions during a period of workforce instability, resulting in the postponement of diagnostic evaluation for several months. During this period of uncertainty, the patient and his family experienced considerable psychological distress due to concern for possible malignancy. Ultimately, a liver biopsy confirmed benign FNH, supported by imaging findings and laboratory evaluation (Table 1) [6].

**Table 1:** Laboratory findings Laboratory evaluation at the time of diagnostic workup, including complete blood count, liver function tests, coagulation profile, metabolic panel, and tumor markers. Liver enzymes and bilirubin levels were within normal limits, and tumor markers, including alpha-fetoprotein (AFP) and protein induced by vitamin K absence or antagonist-II (PIVKA-II), were not elevated, supporting a benign etiology. Values were verified against the original medical records. Overall findings demonstrate normal liver function and non-elevated tumor markers, supporting a benign hepatic process.

Test	Result	Reference Range	Interpretation
WBC (×10³/µL)	5.02	4.0-11.0	Normal
Hemoglobin (g/dL)	15.1	13.5-17.5	Normal
Hematocrit (%)	43.4	41-53	Normal
Platelet (×10³/µL)	166	150-400	Normal
PT (sec)	12.2	11-13.5	Normal
PT (%)	126	70-130	Normal
INR	0.89	0.8-1.2	Normal
aPTT (sec)	32.5	25-35	Normal
Total protein (g/dL)	6.8	6.0-8.3	Normal
Albumin (g/dL)	4.8	3.5-5.0	Normal
Globulin (g/dL)	2	2.0-3.5	Normal
Cholesterol (mg/dL)	187	<200	Normal
Total bilirubin (mg/dL)	0.8	0.1-1.2	Normal
AST (U/L)	35	10-40	Normal
ALT (U/L)	43	7-56	Normal
ALP (U/L)	75	44-147	Normal
Glucose (mg/dL)	110	70-100	Mildly elevated
BUN (mg/dL)	15.5	7-20	Normal
Creatinine (mg/dL)	1.06	0.6-1.3	Normal
eGFR (mL/min/1.73m²)	75.9	>60	Normal
Uric Acid (mg/dL)	5.6	3.5-7.2	Normal
Calcium (mg/dL)	9.5	8.6-10.2	Normal
Phosphorus (mg/dL)	3.5	2.5-4.5	Normal
GGT (U/L)	44	9-48	Normal
AFP (ng/mL)	3.1	<10	Not elevated
PIVKA-II (mAU/mL)	33	<40	Not elevated

Concurrently, in August 2025, the patient presented to the Gwangju Veterans Hospital emergency department with severe right flank pain. Given his aortic dissection history, initial emergency evaluation prioritized excluding vascular complications, including dissection extension or new vascular pathology. After vascular imaging ruled out acute vascular events, subsequent imaging revealed a right upper ureteral stone causing significant obstruction and hydronephrosis. This diagnostic sequence further illustrates how initial anchoring to vascular etiologies influenced early clinical decision-making, delaying recognition of a more common urologic cause of acute flank pain.

**Figure 3:**
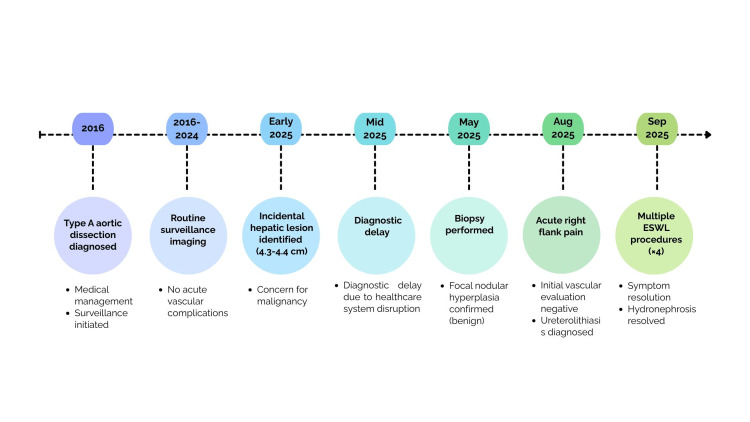
Clinical timeline of a patient with prior type A aortic dissection Key events are arranged chronologically to highlight diagnostic delay and sequential clinical decision-making. The patient was diagnosed with type A aortic dissection in 2016 and managed conservatively with long-term surveillance. In early 2025, routine imaging identified an incidental hepatic lesion, with subsequent diagnostic delay before biopsy confirmed benign focal nodular hyperplasia. In August 2025, the patient presented with acute flank pain and was diagnosed with ureterolithiasis following exclusion of vascular causes, with successful treatment using ESWL. Created by the authors using Canva (Canva Inc., San Francisco, CA).

The patient subsequently required four separate ESWL procedures between August 26 and September 15, 2025, to achieve adequate stone fragmentation and symptom resolution [7]. ESWL was selected as an initial modality, consistent with its non-invasive profile, and the patient's complex vascular history, which raised concerns about more invasive urologic interventions. Stone fragments progressively migrated distally with resolution of hydronephrosis by final follow-up on September 22, 2025. The patient remained hemodynamically stable throughout all procedures with no vascular complications. A chronological overview of the patient’s clinical course is summarized in Figure 3.

Key clinical features in this case that may aid recognition include persistent abdominal or flank pain in a patient with a prior history of major vascular disease, incidental imaging findings suggestive of hepatic pathology, and repeated prioritization of vascular etiologies despite negative vascular workup.

This case further underscores how diagnostic anchoring in high-risk cardiovascular patients can influence acute care decision-making, particularly in emergency and perioperative settings, where rapid exclusion of life-threatening vascular pathology may inadvertently delay consideration of more common alternative diagnoses.

## Discussion

**Figure 4:**
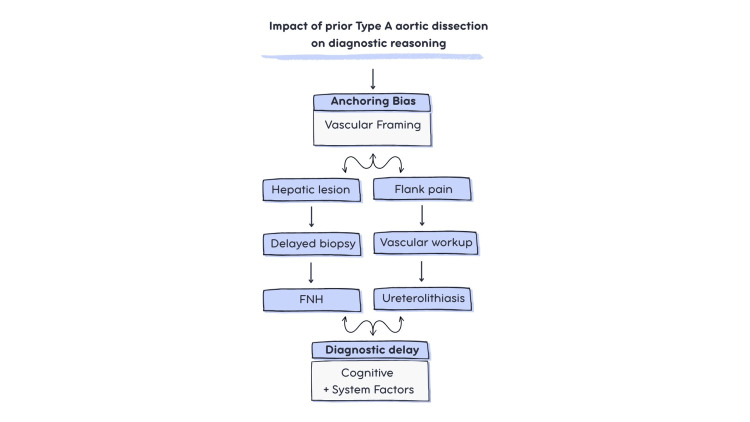
Schematic representation of anchoring bias influencing diagnostic reasoning Schematic illustration of anchoring bias affecting diagnostic pathways in this case. Prior history of type A aortic dissection contributed to cognitive anchoring toward vascular etiologies, influencing evaluation of both an incidental hepatic lesion and acute flank pain. This bias led to delayed recognition of nonvascular conditions, including focal nodular hyperplasia and ureterolithiasis, with additional contribution from system-level factors. Created by the authors using Canva (Canva Inc., San Francisco, CA).

This case illustrates the complex interplay between cognitive bias and system-level factors in delayed diagnosis among aortic dissection survivors. The clinical course appears consistent with anchoring bias at multiple decision points: hepatic mass evaluation was prolonged by initial vascular framing, and acute flank pain triggered reflex exclusion of dissection recurrence before systematic urologic evaluation (Figure 4). These cognitive patterns align with recent studies demonstrating that anchoring bias has been repeatedly identified as a contributor to diagnostic error in emergency and acute care settings [3-5,8]. Importantly, the delays observed here were not solely attributable to diagnostic uncertainty, but to the persistence of vascular-first framing in a survivorship context. These findings support the conclusion that diagnostic delays in this case were multifactorial, arising from both cognitive biases and system-level constraints.

The patient’s prior catastrophic vascular event influenced subsequent clinical reasoning, directing attention toward hemodynamic stability and recurrent dissection. This emphasis contributed to extensive vascular imaging and delayed consideration of ultimately benign hepatic and urologic etiologies.

Anchoring bias in aortic dissection survivors is particularly problematic because the condition's high mortality creates availability bias, in which prior dramatic events increase cognitive accessibility and lead clinicians to overweight vascular diagnoses in subsequent encounters. From a perioperative perspective, heightened vigilance for catastrophic re-dissection is often appropriate in acute settings; however, this risk-weighted cognitive framing may inadvertently influence longitudinal diagnostic reasoning, leading clinicians to emphasize and prioritize vascular etiologies even when more common non-vascular conditions are present. In addition, long-term pharmacologic regimens commonly used in cardiovascular survivorship (e.g., chronic β-blockade, often alongside renin-angiotensin-aldosterone system (RAAS)-modulating agents) can further increase multisystem physiologic complexity and reinforce a “cardiovascular-first” diagnostic frame, particularly when renal or metabolic parameters fluctuate [9].

The cognitive mechanisms underlying this process, including availability bias and premature closure, are reflected in this case. Systematic reviews have identified anchoring bias as a frequent contributor to diagnostic error in emergency and acute care settings [8]. In emergency and critical care settings, anchoring on a patient's past medical history, particularly catastrophic events, can lead to premature closure and failure to adequately consider alternative diagnoses. This case demonstrates that diagnostic anchoring in survivorship care may persist longitudinally beyond the acute event.

The hepatic mass in this case presented diagnostic uncertainty compounded by both cognitive bias and system-level healthcare disruptions. FNH is among the most common benign hepatocellular lesions [6]. While FNH typically demonstrates characteristic imaging features, including arterial phase hyperenhancement and hepatobiliary phase uptake on gadoxetate-enhanced MRI, atypical presentations may occur, necessitating tissue diagnosis in indeterminate cases [10,11]. The patient's vascular history may have influenced the decision-making process, with clinicians potentially anchoring on vascular complications rather than systematically considering benign hepatic pathology.

Similarly, the acute flank pain presentation demonstrates how anchoring bias can delay appropriate diagnostic workup. While vigilance for aortic complications in dissection survivors is appropriate, this must be balanced with consideration of common diagnoses such as nephrolithiasis. Urolithiasis affects approximately 10-15% of the population, with recurrence rates approaching 50% over 10 years [7]. The patient's requirement for multiple ESWL sessions aligns with contemporary data showing that stone-free rates after the first ESWL session range from 46.7% to 69%, with repeated treatments often necessary to achieve optimal outcomes [7]. Although vascular complications warrant prompt exclusion, the initial “vascular-first” framing can still distort probabilistic reasoning when alternative diagnoses are common [3-5,8].

System-level factors may also have contributed to the prolonged diagnostic interval. The patient’s evaluation occurred during a period of healthcare workforce instability in South Korea, which has been associated with delays in surgical and diagnostic services [12]. While direct causation cannot be established in this single case, such system strain may have compounded existing diagnostic uncertainty.

Several authors have proposed structured differential diagnosis checklists and cognitive forcing strategies to mitigate anchoring bias in high-risk settings [4,5,8,13,14]. Cognitive forcing strategies include deliberate reconsideration of alternative diagnoses prior to closure and structured use of diagnostic checklists to reduce premature closure and anchoring effects [13,14]. First, the use of structured differential diagnosis checklists may encourage clinicians to engage in deliberate rather than reflexive reasoning [4,5]. Second, cognitive forcing techniques like "What else could it be?" and "What are we missing?" may stimulate active thinking mechanisms against the anchoring effect. Third, consultation with clinicians not influenced by the initial diagnostic framing may be beneficial.

From a surgical and perioperative perspective, diagnostic anchoring in patients with a history of catastrophic vascular disease presents unique clinical challenges. Surgeons and acute care physicians are trained to prioritize life-threatening vascular complications when evaluating new symptoms in these patients. While this vigilance is appropriate in emergency settings, it may unintentionally bias subsequent diagnostic reasoning, particularly when patients present with non-specific abdominal or flank pain. A structured diagnostic framework that combines early exclusion of catastrophic vascular pathology with parallel consideration of common etiologies may help mitigate this risk. Multidisciplinary collaboration among vascular surgeons, radiologists, hepatologists, and urologists may also improve diagnostic efficiency and reduce unnecessary delays in care.

This case has several limitations. First, the diagnostic delays were multifactorial, involving both cognitive biases and system-level healthcare disruptions, making it difficult to quantify the relative contribution of each factor. Second, retrospective case analyses are subject to hindsight bias, and what appears as anchoring bias in retrospect may have represented appropriate clinical vigilance at the time. Finally, this single case cannot establish causation between cognitive biases and diagnostic delays, though it illustrates patterns consistent with well-documented cognitive phenomena. The broader clinical, system-level, and educational implications derived from this case are discussed above.

Future directions and ideas for further research include the development of a global survey protocol to study inherent bias and predisposition of clinicians toward diagnostic anchoring with key medical conditions that put patients at high risk of death. Such a study would encompass resident physicians as well as attending/consultant surgeons for a balanced perspective. This issue remains underexplored in the literature and especially lacks longitudinal prospective studies to establish pathways for solutions and education for the medical community.

## Conclusions

This case illustrates how cognitive anchoring bias and system-level disruptions in healthcare can combine to produce significant diagnostic delays among aortic dissection survivors. The diagnostic delay observed in this case was multifactorial, reflecting both cognitive biases (anchoring, availability, and premature closure) and external system constraints. Clinicians must balance appropriate vigilance for vascular complications with systematic consideration of common non-vascular diagnoses. Implementation of structured diagnostic approaches, cognitive forcing strategies, and multidisciplinary consultation may help mitigate anchoring bias and improve diagnostic accuracy in this high-risk population. Healthcare systems must also ensure contingency planning to maintain essential diagnostic services during periods of disruption. Survivors of major vascular catastrophes represent a population vulnerable to longitudinal diagnostic anchoring, and structured cognitive checkpoints may reduce delayed recognition of non-vascular pathology in this group.
